# The First Genetic Characterization of the *SPRN* Gene in Pekin Ducks (*Anas platyrhynchos domesticus*)

**DOI:** 10.3390/ani14111588

**Published:** 2024-05-27

**Authors:** Thi-Thuy-Duong Nguyen, Mohammed Zayed, Yong-Chan Kim, Byung-Hoon Jeong

**Affiliations:** 1 Korea Zoonosis Research Institute, Jeonbuk National University, 820-120, Hana-ro, Iksan 54531, Republic of Koreamzayed2@vet.svu.edu.eg (M.Z.); 2 Department of Bioactive Material Sciences, Institute for Molecular Biology and Genetics, Jeonbuk National University, Jeonju 54896, Republic of Korea; 3 Department of Surgery, College of Veterinary Medicine, South Valley University, Qena 83523, Egypt; 4 Department of Biological Sciences, Andong National University, Andong 36729, Republic of Korea

**Keywords:** pekin ducks, prion, *SPRN*, Sho, genetic polymorphism, SNP

## Abstract

**Simple Summary:**

The shadow of prion protein (Sho) encoded by the shadow of prion protein gene (*SPRN*) has been implicated in prion disease biology. Here, we identified the *SPRN* gene sequence and characterized its genetic polymorphisms in ducks. As a result, the duck and chicken Sho amino acid sequences shared 100% identity. Notably, we found an abundance of single nucleotide polymorphisms in the open reading frame of duck *SPRN* gene, which is comparable to those in prion disease-susceptible species. To date, there has been no evidence of prion disease in ducks to our knowledge. Given that our previous study on the duck prion protein gene suggested several characteristics of potential prion disease susceptibility, this study supports the need for additional experiments to examine the risk of developing prion diseases in ducks.

**Abstract:**

Prion diseases are fatal neurodegenerative disorders characterized by an accumulation of misfolded prion protein (PrP^Sc^) in brain tissues. The shadow of prion protein (Sho) encoded by the shadow of prion protein gene (*SPRN*) is involved in prion disease progress. The interaction between Sho and PrP accelerates the PrP^Sc^ conversion rate while the *SPRN* gene polymorphisms have been associated with prion disease susceptibility in several species. Until now, the *SPRN* gene has not been investigated in ducks. We identified the duck *SPRN* gene sequence and investigated the genetic polymorphisms of 184 Pekin ducks. We compared the duck *SPRN* nucleotide sequence and the duck Sho protein amino acid sequence with those of several other species. Finally, we predicted the duck Sho protein structure and the effects of non-synonymous single nucleotide polymorphisms (SNPs) using computational programs. We were the first to report the Pekin duck *SPRN* gene sequence. The duck Sho protein sequence showed 100% identity compared with the chicken Sho protein sequence. We found 27 novel SNPs in the duck *SPRN* gene. Four amino acid substitutions were predicted to affect the hydrogen bond distribution in the duck Sho protein structure. Although MutPred2 and SNPs&GO predicted that all non-synonymous polymorphisms were neutral or benign, SIFT predicted that four variants, A22T, G49D, A68T, and M105I, were deleterious. To the best of our knowledge, this is the first report about the genetic and structural characteristics of the duck *SPRN* gene.

## 1. Introduction

Prion diseases are fatal neurodegenerative diseases that affect many species, including humans. They can be inherited, acquired, or occur spontaneously [1]. The pathogenesis of prion diseases is characterized by the conversion of the normal form of the prion protein (PrP^C^) into a partially protease-resistant, misfolded form of prion protein (PrP^Sc^) and the progressive accumulation of this deleterious form in the central nervous system [1]. Recent evidence suggests that the prion protein (PrP) plays a central role in the prion disease process, as its expression is essential for the pathogenesis of prion diseases [2,3]. PrP-deficient mice are resistant to prion infection, and the expression level of PrP correlates with the incubation time of prion infection [4,5,6]. However, the physiological functions of PrP^C^ and its association with additional factors in disease progression remain largely undetermined. The shadow of prion protein gene (*SPRN*), which encodes the shadow of prion protein (Sho), is a member of the prion protein family and is widely conserved from zebrafish to mammals [7]. Sho shares several similarities with PrP, including an N-terminal signal sequence, a repeat region, a hydrophobic domain, and a C-terminal signal sequence for glycophosphatidylinositol anchor attachment [8,9]. The *SPRN* gene is mainly expressed in brain tissues, the most important organ for prion infection, and it displays PrP^C^-like neuroprotective activity [10,11]. 

A positive correlation between the mRNA expression levels of the prion protein gene (*PRNP*) and *SPRN* genes indicates co-regulation between them [12]. Although several studies have not supported a direct causative role for Sho in prion infection [13,14], Sho is still interesting as a crucial factor in prion biology for the following reasons: (1) the downregulation of Sho in prion-infected rodents and neuroblastoma cells, but not in mice with other neurodegenerative diseases, and its correlation with levels of infectious prions in the preclinical phase suggests that it could be a non-PrP-specific marker for prion infection [10,15,16,17,18]; (2) the interaction between Sho and PrP modifies the PrP-folding pathway and could contribute to an increased conversion rate from PrP^C^ to PrP^Sc^ [19]; (3) the interaction site between Sho and PrP^C^ in the hydrophobic domain, which is the most homologous region of these two protein sequences, suggests that Sho plays a role in the physiological functions of PrP^C^ and prion pathogenesis [20]. 

In humans, single nucleotide polymorphisms (SNPs) at codons 129 and 219 of the *PRNP* gene affect susceptibility to Creutzfeldt–Jakob disease (CJD) [21,22,23,24]. Moreover, previous studies have reported that non-synonymous SNPs, such as S167D in the horse and N159D in the dog *PRNP* gene, induce different phenotypes in degenerating brain neurons and result in decreased β-sheet formation, contributing to resistance to prion disease [25]. Beyond the association between *PRNP* polymorphisms and the risk of developing prion diseases, several findings suggest that other genes might also affect prion disease susceptibility. For example, Beck et al. demonstrated a significant association between a null allele in the *SPRN* gene and the occurrence of variant CJD within the British population [26]. Additionally, investigations have revealed that an insertion/deletion (indel) polymorphism in the 3′ untranslated regions (UTRs) and another in the open reading frame (ORF) are linked to susceptibility to scrapie in goat [27] and sheep [28], respectively.

Therefore, further studies on the *SPRN* gene might give more insight into prion biology. Prion diseases have been reported in a wide range of species, including CJD in humans, bovine spongiform encephalopathy in cattle, scrapie in sheep, chronic wasting disease in the Cervidae family, and feline spongiform encephalopathy in cats [29]. However, they have not yet been reported in birds [30,31]. Pekin duck (*Anas platyrhynchos domesticus*) is the main breed raised by commercial producers and comprises a large portion of the poultry meat market, especially in Asian countries [32]. The demand for this meat is increasing moderately, leading to a rise in duck numbers globally. It has been reported that duck PrP displays a higher β-sheet structure, which is proposed to affect the conversion of PrP^C^, and that it has a higher aggregation propensity than chicken PrP [33]. These features suggest the need for further studies to characterize other genes that might be associated with prion disease susceptibility in ducks. To date, the duck *SPRN* gene has not been studied; thus, it is necessary to investigate the duck *SPRN* gene to elucidate its prion-related characteristics. 

In this work, we aimed to identify the duck *SPRN* gene sequence and compare its genetic characteristics with those of other species. We also investigated genetic polymorphisms in the duck *SPRN* gene. In addition, we analyzed the linkage disequilibrium (LD) and the genotype, allele, and haplotype frequencies of the identified SNPs. Furthermore, we studied the effects of the identified SNPs on the predicted three-dimensional (3D) structure of the duck Sho protein.

## 2. Materials and Methods

### 2.1. Ethical Statement

Brain tissues from Pekin ducks (*n* = 184) were collected from Harim, Inc., which is the largest food company in Korea and sources ducks from regional farms across the country. The experimental procedures were approved by the Institutional Animal Care and Use Committee (IACUC) of Jeonbuk National University (IACUC number: JBNU2017-0030).

### 2.2. Genetic Analysis of Pekin Duck SPRN

DNA was extracted from brain tissues using a Labopass tissue genomic DNA isolation kit (Cosmo Genetech Co., Ltd., Seoul, Republic of Korea) according to the manufacturer’s instructions. To amplify the duck *SPRN* gene, we performed polymerase chain reaction (PCR) using forward primer F: 5′-CTCCCTGTGTGCAGGTCAG-3′ and reverse primer R: 5′-TACATGTATCCCTGCGCCTG-3′, which were designed based on the chicken *SPRN* gene (Gene ID: BN000836.1).

The PCR mixture included 2 μL of genomic DNA, 10 pmol of each primer, 2.5 μL of 10× H-star *Taq* reaction buffer, 1 μL of dNTP 10mM mixture, 2.5 μL of 5× Band Helper, 0.2 μL of H-star *Taq* DNA polymerase (2.5 U/μL) (BioFact^TM^, Daejeon, Republic of Korea), and distilled water to make a final volume of 25 μL. The PCR conditions were as follows: initial denaturing at 98 °C for 15 min; 40 cycles of 98 °C for 20 s, 56 °C for 40 s, and 72 °C for 1 min; and 1 cycle of 72 °C for 5 min for final extension. The PCR products were confirmed through gel electrophoresis, purified using a FavorPrep GEL/PCR purification mini kit (FAVORGEN, Ping Tung, Taiwan), and then sequenced using an ABI 3730 sequencer (ABI, Foster City, CA, USA). We used Finch TV software version 1.4.0 (Geospiza Inc., Seattle, WA, USA) to visualize the sequencing results.

### 2.3. Multiple Sequence Alignment

Amino acid sequences of the Sho protein from several species were aligned using Clustal Omega https://www.ebi.ac.uk/Tools/msa/clustalo/ (assessed date 21 April 2023). Amino acid sequences were obtained from GenBank, National Center for Biotechnology Information. Detailed information is provided in Supplementary Table S1.

### 2.4. 3D Structure Prediction of Duck Sho Protein

AlphaFold2 was used to generate the 3D structure of the duck Sho protein https://colab.research.google.com/github/sokrypton/ColabFold/blob/main/AlphaFold2.ipynb (assessed date 30 August 2023). AlphaFold2 is a highly accurate protein structure prediction program developed by DeepMind. We further visualized the predicted model with the SWISS-Pdb Viewer program version 4.1.0, http://spdbv.vital-it.ch/ (assessed 3 May 2023) to evaluate the effects of amino acid substitutions on the 3D structure of the duck Sho protein, primarily by examining changes in hydrogen bonds. For validation, we used the SWISS-MODEL program, which is one of the most widely used web-based prediction tools, with the A2BDG0.1.A template [34]. 

### 2.5. In Silico Evaluation of the Effect of Non-Synonymous SNPs on the Duck Sho Protein 

To analyze the effects of non-synonymous SNPs on duck Sho, we used the Sorting Intolerant Form Tolerant (SIFT) https://sift.bii.a-star.edu.sg/www/SIFT_aligned_seqs_submit.html (assessed date 23 May 2023), MutPred2 http://mutpred.mutdb.org/ (assessed date 30 August 2023), and SNPs&GO http://snps.biofold.org/snps-and-go (assessed date 24 May 2023) programs. SIFT predicts the deleterious effects of a non-synonymous SNP on protein function by using sequence homology. SIFT predicts substitutions with scores of less than 0.05 as deleterious [35]. It requires an aligned-sequence database, which we created using the duck Sho protein sequence as a query in a PSI-BLAST search on a non-redundant sequence database downloaded from GenBank (assessed date 22 May 2023) that consists of 544,417,592 sequences, with a PSI-BLAST threshold of 0.001. To prevent the overrepresentation of specific sequences, only sequences found using PSI-BLAST with an e-value < 0.001 and an identical percent between 35% and 90% were retained after each search and used for the next iteration [36]. MutPred2 is a machine learning-based program that integrates genetic and molecular data to estimate the pathogenicity of amino acid substitutions. A score equal to or higher than 0.5 indicates pathogenicity [37]. SNPs&GO determines the probability that each single amino acid polymorphism will be associated with disease by using a support vector machine to calculate the probability of pathogenicity based on functional information codified by Gene Ontology. The threshold of 0.5 indicates that a variation is disease-related [38].

### 2.6. Statistical Analysis

The Hardy–Weinberg equilibrium (HWE), haplotypes, and LD analyses were conducted on the genotyping data of all SNPs using Haploview version 4.2 (Broad Institute, Cambridge, MA, USA) [39]. The HWE test was performed to assess whether the observed genotypes conform to the HWE expectation. The LD analysis was calculated using the pairwise LD (*r*^2^), and strong LD was identified when *r*^2^ > 0.333 [40].

## 3. Results

### 3.1. Identification of the SPRN Gene Sequence of Pekin Ducks 

**Figure 1:**
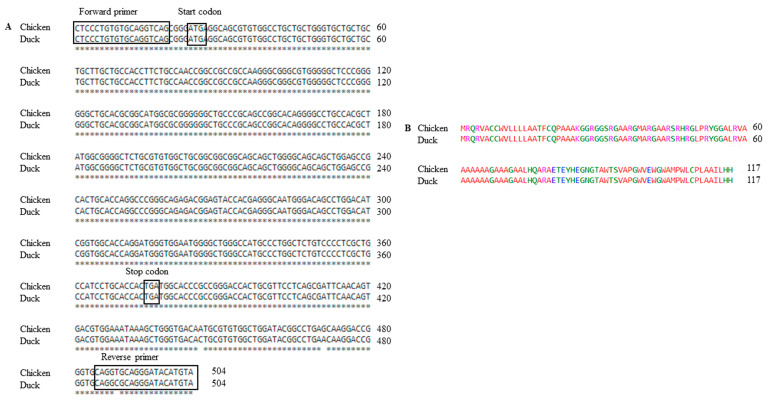
Identification of the shadow of prion protein gene (*SPRN*) in Pekin ducks: (**A**) Comparison of the *SPRN* gene sequences between chicken (*Gallus gallus* BN000836.1) and Pekin duck (this study). Nucleotide sequences were aligned using Clustal Omega https://www.ebi.ac.uk/Tools/msa/clustalo/ (assessed date 21 April 2023). Asterisks indicate identical nucleotides between the chicken and duck *SPRN* genes. (**B**) Sequence alignment between duck and chicken Sho proteins. Colors indicate the chemical properties of the amino acids: blue, acidic; red, small and hydrophobic; magenta, basic; green, hydroxyl + sulfhydryl + amine and glycine.

We designed primers based on the chicken *SPRN* gene sequence (Gene ID: BN000836.1) to obtain an amplicon of the duck *SPRN* gene encompassing the entire ORF and a small fragment of the 3′ UTR. The alignment of the identified duck *SPRN* gene sequence with the chicken *SPRN* gene sequence registered in GenBank revealed only three mismatches, with 99.4% sequence homology (501/504) (Figure 1).

### 3.2. Comparison of the Duck Sho Protein Sequence with Those of Several Species 

**Figure 2:**
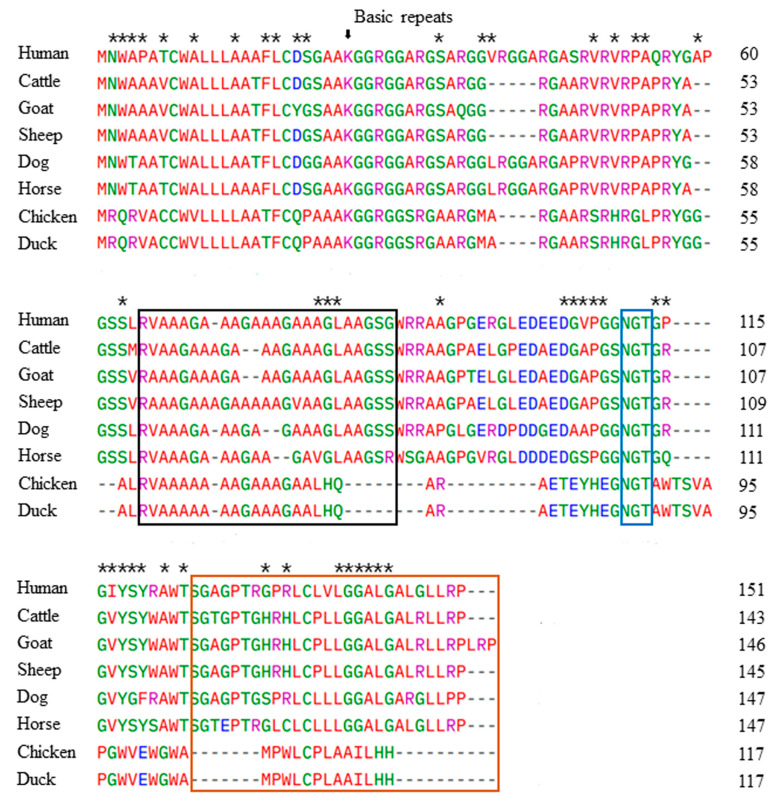
Amino acid sequence alignments of the Sho protein in humans, cattle, goats, sheep, dogs, horses, chickens, and ducks. Colors indicate the chemical properties of the amino acids: blue, acidic; red, small and hydrophobic; magenta, basic; green, hydroxyl + sulfhydryl + amine and glycine. Asterisks indicate duck-specific amino acids, and the arrow indicates the beginning of the basic region. Black box: a hydrophobic alanine-rich sequence, which is an indispensable factor for PrP^C^/PrP^Sc^ interaction with Sho (the most homologous region of Sho and PrP); blue box: glycosylation motif; red box: signal sequence for the GPI anchor of Sho.

We performed multiple sequence alignments of the Sho protein from several species, including prion disease-susceptible and -resistant species, using Clustal Omega. The results show that the amino acid sequence of the duck and chicken Sho protein is shorter (117 amino acids) than the Sho protein in other species (Figure 2). The amino acid sequence of the duck Sho has 100% identity with that of chicken, followed by human (49.57%), dog (47.86%), horse (47.01%), sheep (47.01%), goat (46.15%), and cattle (45.3%). The major structural features of Sho were conserved across the investigated species, including the basic R-G-type repeats (indicated by an arrow at the start region) and the glycosylation motif site (highlighted in a blue box) (Figure 2). Although the N-terminal signal peptide of mammals exhibited high conservation among species, it differed from that of the avian Sho. Additionally, the alanine-rich fragment (marked by a black box and representing the hydrophobic alanine region), which is homologous between Sho and PrP, showed variations among the investigated species, especially between prion disease-susceptible and prion disease-resistant animals. Notably, the chicken and duck Sho sequences consist of 47 amino acids, which differ from the other species investigated in this study (Figure 2).

### 3.3. Identification of Novel SNPs for the SPRN Gene in Pekin Ducks

**Figure 3:**
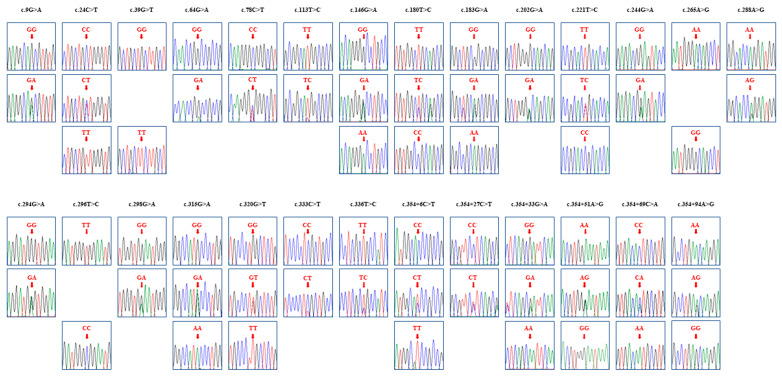
The electropherograms of the 27 novel single nucleotide polymorphisms (SNPs) found in the duck shadow of prion protein (*SPRN*) in this study. The colors of the peaks indicate each base of the DNA sequence (green: adenine; red: thymine; blue: cytosine; and black: guanine). The red arrows indicate the locations of SNPs found in this study.

Genotyping of the duck *SRPN* gene in a sample from 184 ducks was performed using the sequencing data for the PCR products. We identified 27 novel SNPs, including nine synonymous variants at positions c.9G>A, c.24C>T, c.39G>T, c.78C>T, c.180T>C, c.183G>A, c.288A>G, c.333C>T, and c.336T>C. Interestingly, there were twelve non-synonymous SNPs, at positions c.64G>A (A22T), c.113T>C (M38T), c.146G>A (G49D), c.202G>A (A68T), c.221T>C (L74P), c.244G>A (E82K), c.265A>G (T89A), c.294G>A (W98X), c.296T>C (V99A), c.298G>A (E100K), c.315G>A (M105I), and c.320G>T (W107L). In addition, six SNPs were located in the 3′UTR, specifically at positions c.354+6C>T, c.354+27C>T, c.354+33G>A, c.354+51A>G, c.354+69C>A, and c.354+94A>G. Electropherograms displaying the 27 SNPs are presented in Figure 3. 

The genotype and allele frequencies for the 27 SNPs in this study are comprehensively described in Table 1. Subsequently, we calculated the LD score among all SNPs of the duck *SPRN* gene, revealing fourteen strong linkages (*r*^2^ > 0.333) (Table 2). Table 3 presents the twelve major haplotypes with proportions higher than 1%. The haplotype GCGGCTGTGGTGAAGTGGGCTCCGACA had the highest proportion, accounting for 24.3%, and it was followed by GCGGCTGTAGTGAAGTGGGCTCCGACA at 13.6%.

### 3.4. Effects of Amino Acid Substitutions on the 3D Structure of the Duck Sho Protein 

**Figure 4:**
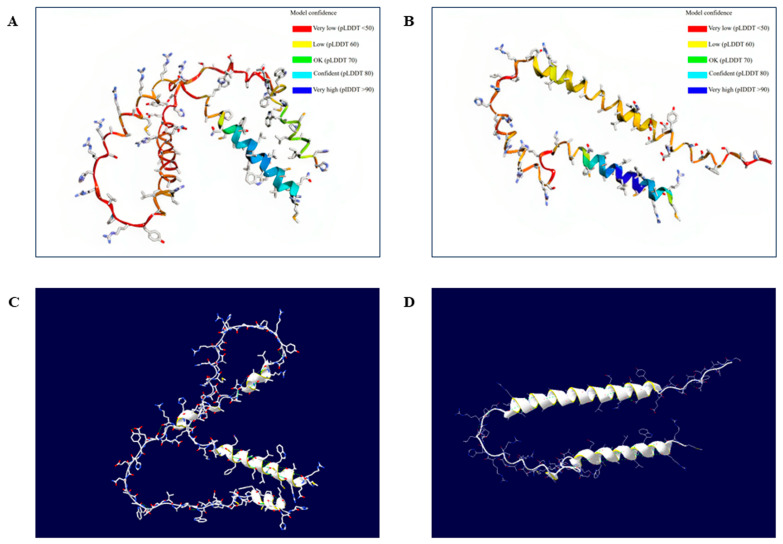
Tertiary structure of the Sho protein in Pekin ducks: (**A**) The tertiary structure of the duck Sho protein with the wild-type allele predicted using AlphaFold2. pLDDT, predicted local distance different test. (**B**) The tertiary structure of the duck Sho protein with the nonsense polymorphism at codon 98 predicted using AlphaFold2. (**C**) Analysis of hydrogen bonds in the duck Sho protein with the wild-type allele, visualized using SWISS-Pdb Viewer. Green dotted lines indicate hydrogen bonds. Helix ribbons are displayed in white and yellow. (**D**) Analysis of hydrogen bonds in the duck Sho protein with the nonsense polymorphism at codon 98, visualized using SWISS-Pdb Viewer. Green dotted lines indicate hydrogen bonds. Helix ribbons are displayed in white and yellow.

**Figure 5:**
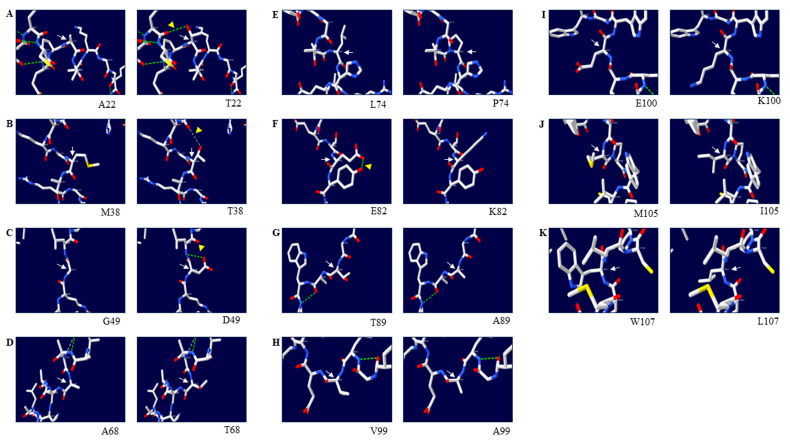
Prediction of the tertiary structure and hydrogen bonds of the shadow of prion protein (Sho) in ducks: (**A**) Comparison of the 3D structure and hydrogen bonds of the duck Sho according to the A22T allele. (**B**) Comparison of the 3D structure and hydrogen bonds of the duck Sho according to the M38T allele. (**C**) Comparison of the 3D structure and hydrogen bonds of the duck Sho according to the G49D allele. (**D**) Comparison of the 3D structure and hydrogen bonds of the duck Sho according to the A68T allele. (**E**) Comparison of the 3D structure and hydrogen bonds of the duck Sho according to the L74P allele. (**F**) Comparison of the 3D structure and hydrogen bonds of the duck Sho according to the E82K allele. (**G**) Comparison of the 3D structure and hydrogen bonds of the duck Sho according to the T89A allele. (**H**) Comparison of the 3D structure and hydrogen bonds of the duck Sho according to the V99A allele. (**I**) Comparison of the 3D structure and hydrogen bonds of the duck Sho according to the E100K allele. (**J**) Comparison of the 3D structure and hydrogen bonds of the duck Sho according to the M105I allele. (**K**) Comparison of the 3D structure and hydrogen bonds of the duck Sho according to the W107L allele. The left panes indicate the 3D structure of the duck Sho with the wild-type allele, and the right panels indicate the 3D structure of the duck Sho with the substitution at the indicated position. Green dotted lines indicate strong hydrogen bonds, gray dotted lines indicate weak hydrogen bonds, and purple dotted lines indicate clash bonds. The green numbers indicate the distance of the hydrogen bonds. White arrows indicate the alleles’ position. Yellow triangles indicate H-bond changes.

To examine the effects of non-synonymous SNPs on the duck Sho protein, we initially generated a 3D structural model of the duck Sho using AlphaFold2. Subsequently, we used the Swiss-Pdb Viewer program to observe changes in the 3D structure of duck Sho with a nonsense mutation at codon 98 (Figure 4). Meanwhile, SWISS-MODEL was conducted to validate the predicted results. The models predicted by two web-based tools showed similar structures in the N- and C-terminals with high confidence scores (Figure S1). Since AlphaFold2 represents the highest accuracy compared to other programs, regardless of the prediction methods [41], the 3D structural model predicted using AlphaFold2 was used for downstream analysis. The effects of the individual non-synonymous SNPs were analyzed by investigating changes in hydrogen bonding (Figure 5). The findings reveal that c.294G>A (W98X) induces premature termination of translation for the duck *SPRN* gene, leading to the generation of a truncated variant of the Sho protein. This truncation introduces two novel alpha-helical structures into the duck Sho protein. Furthermore, distinctions in hydrogen bonds were observed with respect to genetic alleles at codons 22, 38, 49, and 82. Specifically, at codon 22, the A22 allele was computationally predicted to have no hydrogen bond (Figure 5A, left panel). In contrast, the T22 allele was projected to establish a hydrogen bond with C18 (3.29 Å). At codon 38, T38 was anticipated to create a weak hydrogen bond with A35 (3.32 Å). Similarly, the D49 residue was predicted to form a hydrogen bond with L50 (3.18 Å), whereas the G49 residue did not. At codon 82, E82 was computationally forecast to engage in a hydrogen bonding interaction with Y83 (2.98 Å); however, K82 was not expected to form such a bond.

### 3.5. In Silico Evaluation of the Effects of Non-Synonymous SNPs on the Duck Sho Protein

The predicted effects of the protein variations are summarized in Table 4. Notably, both MutPred2 and SNPs&GO predicted that all the non-synonymous SNPs for the duck Sho protein would have neutral effects. However, SIFT predicted deleterious effects for the non-synonymous SNPs at codons 22, 49, 68, and 105.

### 3.6. Distribution of SNPs in the ORF of the SPRN Gene in Several Animals 

**Figure 6:**
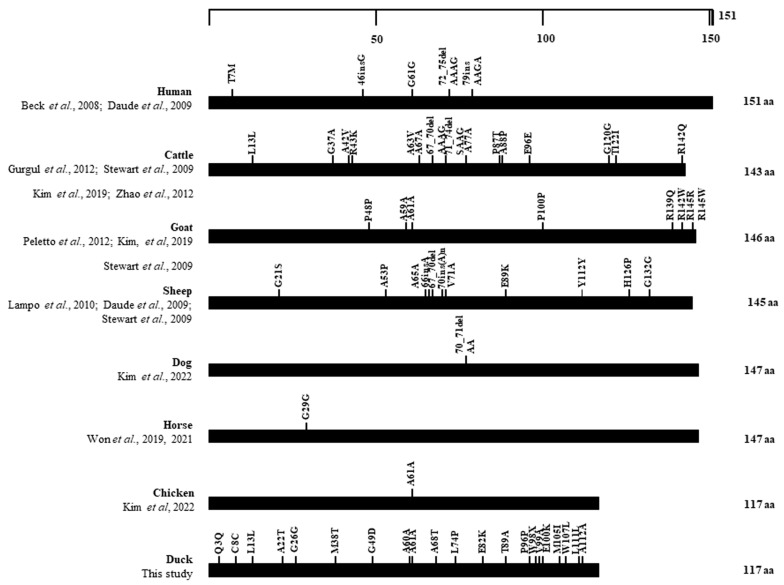
Distribution of genetic polymorphisms in the open reading frame (ORF) of the *SPRN* gene in various species. The figure shows previously reported genetic polymorphisms of the *SPRN* gene in humans [26,42], cattle [15,43,44,45], goats [27,44,46], sheep [28,42,44], dogs [47], horses [48,49], chickens [50], and ducks (this study). The edged horizontal bar indicates the number of amino acids in the *SPRN* gene.

We collected reported genetic polymorphisms in the ORF of the *SPRN* gene in prion disease-susceptible animals (humans [26,42], cattle [15,43,44,45], sheep [28,42,44], goats [27,44,46]), and prion disease-resistant animals (dogs [47], horses [48,49], chickens [50], and ducks (this study) to compare the distribution of SNPs between these two groups (Figure 6). We noticed that various SNPs were found in the ORF of the prion disease-susceptible group, whereas, in the prion disease-resistant group, except for ducks, only one SNP was found in the ORF of the *SPRN* gene.

## 4. Discussion

The *SPRN* gene plays an important role in prion diseases. In this study, we successfully amplified the duck *SPRN* gene for the first time. The identified *SPRN* gene sequence of the Pekin duck displays 99.4% sequence homology with the chicken *SPRN* gene sequence. Remarkably, we also observed 100% identity between the predicted ORF of the *SPRN* gene in ducks and chickens. Our multiple alignment results for Sho amino acid sequences among several species confirm the main features of the duck Sho protein, including an N-terminal sequence, a basic R-G-rich region, a hydrophobic domain, a C-terminal region with an NGT motif, and a possible anchor site. This result is consistent with the study of Premzl et al. from 2003 [7]. The R-G-rich region containing tetra-repeats of the consensus X-X-R-G and the N-glycosylation site was highly conserved among all species, from mammals to birds [7]. However, the hydrophobic region of the Sho protein differed between the prion disease-susceptible and prion disease-resistant animals. Previous studies have reported that the homologous region between Sho and PrP serves as the interaction site between the two proteins [20], and that the deletion of this site in the Sho protein causes the loss of its stress-protective activity [11]. Modifications in the highly conserved R-G-rich region and hydrophobic domain might hold functional importance and affect prion disease susceptibility. 

The *SPRN* gene is one of the key factors in prion disease biology through its role in promoting the conversion from PrP^C^ to PrP^Sc^. Thus, genetic polymorphisms of the *SPRN* gene are significantly related to susceptibility to prion diseases and could affect the function and structure of the Sho protein. In this work, we identified 27 novel SNPs. Notably, four of them, c.183G>A, c.354+33G>A, c.354+69C>A, and c.354+94A>G, are homologous to four SNPs identified in the chicken *SPRN* gene in a previous study [50], suggesting conservation among birds’ *SPRN* genes. 

The effects of the variants on Sho biology remain unclear; however, the number and position of the variants could provide insight into their likely functionality. Therefore, we investigated the distribution of genetic polymorphisms in the ORF of the *SPRN* gene in several species. Interestingly, we observed that only one polymorphism was found in horses, dogs, and chickens—prion disease-resistant animals—whereas abundant SNPs were found in prion disease-susceptible animals. This observation supports the hypothesis that the absence of polymorphisms in the coding region of the *SPRN* gene could be unique to prion disease-resistant animals [49,50]. Nonetheless, Pekin ducks, which have been considered a prion disease-resistant species, display a variety of polymorphisms in the ORF of the *SPRN* gene. Additionally, Jeong et al. pointed out other features related to prion diseases in ducks, noting that the PrP of Pekin ducks showed a high proportion of β-sheets and a high aggregation propensity [33]. This study, however, was unable to determine whether the duck *SPRN* gene is expressed as a protein. Future works are required to address this limitation and further evaluate the features identified in the duck *SPRN* gene and the duck *PRNP* gene, which might be present in prion disease-susceptible species. Understanding the features underlying susceptibility and resistance to prion diseases could contribute to therapeutic development and disease prevention [51]. Moreover, ducks play an important role in meat production and the transmission of several infectious diseases [52,53]. Even though no cases of prion disease have been reported in ducks to our knowledge, it is necessary to validate the possibility of developing transmissible spongiform encephalopathies in ducks via experimental studies. 

We did not identify any indel variants in the investigated region. Previous association studies have revealed frameshift polymorphisms associated with scrapie in sheep [27] and variant CJD in humans [26]. In particular, a variety of indel polymorphisms in the Ala-rich segment, including the insertion of a chain of Ala at codon 65 and a deletion of four amino acids AAAG (codons 67–70) in sheep [27,42,44] and cattle [15,28,43,45], cause instability in the hydrophobic domain. This finding might be a special feature of the ovine and bovine genes, contributing to species differences. In addition, Zhao and Liu reported a 12-bp indel polymorphism in the hydrophobic domain of cattle—a prion disease-susceptible animal—but not in buffalo—a prion disease-resistant ruminant—suggesting that this frameshift variant might influence susceptibility or resistance to prion infection in domestic bovids [15]. Thus, the absence of indel polymorphisms but an abundance of SNPs might be a unique feature of the duck *SPRN* gene. 

Next, we estimated the effects of amino acid substitutions on the duck Sho protein using *in silico* programs. Of note, SIFT predicted that four amino acid substitutions (A22T, G49D, A68T, and M105I) were deleterious (Table 4), suggesting that those SNPs might affect Sho protein function. SIFT predicts whether an amino acid substitution is functionally neutral or deleterious based on sequence homology and the amino acids’ physical properties. The increase in PrP folding induced by interaction between Sho and PrP suggests that Sho has a role in prion disease pathogenicity [19]. Any changes in the Sho protein caused by a deleterious amino acid substitution might thus affect susceptibility to prion diseases. Further experimental evaluation in a future study is needed to understand how amino acid substitutions affect the duck Sho protein. 

We also predicted the 3D structure of the duck Sho protein to evaluate the effects of amino acid substitutions. By comparing the 3D structure of the duck Sho protein with the wild-type allele and a nonsense variant at codon 98 predicted using AlphaFold2, we identified that this polymorphism results not only in an incomplete protein sequence but also an increase in the number of alpha helices (Figure 4). Several studies have suggested that the truncated version of the PrP protein might fail to bind properly to the plasma membrane, leading to variations in disease duration or the phenotypes of prion diseases [54,55]. However, the effects of this nonsense variant on Sho function and the prion disease susceptibility of ducks still require further examination in the future. 

Hydrogen bonds can contribute to the stability and structure of proteins [56,57]. Therefore, we compared the hydrogen bond distribution and the distance of hydrogen bonds between the wild-type and missense alleles of the duck Sho protein. We observed hydrogen bond distribution changes with four SNPs: A22T, M38T, G49D, and E82K (Figure 5). Interestingly, two of those SNPs (A22T, G49D) were also predicted using SIFT to have deleterious effects on the protein.

However, our study was limited to addressing the exon structure of the duck *SPRN* gene. Future works, including rapid amplification of cDNA ends (RACE) sequencing, are needed to validate the complete genomic information of the duck *SPRN* gene. 

## 5. Conclusions

It is clear that genes other than *PRNP*, such as the *SPRN* gene, play a role in prion disease progress. In this work, we amplified and identified the duck *SPRN* gene sequence. Notably, the duck Sho protein sequence shows 100% identity with the chicken Sho protein. In addition, we found 27 novel SNPs in the duck *SPRN* gene. We predicted the 3D structure of the duck Sho protein and observed hydrogen bond changes in its 3D structure as consequences of the amino acid substitutions we found. Finally, we predicted the effects of non-synonymous SNPs on the duck Sho protein using three *in silico* programs. The results predicted using SIFT partially align with the results obtained by evaluating hydrogen bond changes. To the best of our knowledge, this has been the first study on the duck *SPRN* gene.

## Figures and Tables

**Table 1:** Genotype and allele frequencies of the single nucleotide polymorphisms in the shadow of prion protein gene (*SPRN*) in Pekin ducks.

Polymorphisms	Genotype Frequencies; *n* (%)			Allele Frequencies; *n* (%)		HWE *p*-Value
	M/M	M/m	m/m	M	m	
c.9G>A (Q3Q)	84 (45.65)	100 (54.35)	0 (0.0)	268 (72.8)	100 (27.2)	<0.001
c.24C>T (C8C)	166 (90.22)	17 (9.24)	1 (0.54)	349 (94.8)	19 (5.2)	0.7741
c.39G>T (L13L)	183 (99.46)	0 (0.0)	1 (0.54)	366 (99.5)	2 (0.5)	0.0054
c.64G>A (A22T)	162 (88.04)	22 (11.96)	0 (0.0)	346 (94.0)	22 (6.0)	1.0
c.78C>T (G26G)	181 (98.37)	3 (1.63)	0 (0.0)	365 (99.2)	3 (0.8)	1.0
c.113T>C (M38T)	181 (98.37)	3 (1.63)	0 (0.0)	365 (99.2)	3 (0.8)	1.0
c.146G>A (G49D)	99 (53.8)	82 (44.57)	3 (1.63)	280 (76.1)	88 (23.9)	0.0024
c.180T>C (A60A)	161 (87.5)	22 (11.96)	1 (0.54)	344 (93.5)	24 (6.5)	1
c.183G>A (A61A)	54 (29.35)	117 (64.13)	12 (6.52)	226 (61.4)	142 (38.6)	<0.001
c.202G>A (A68T)	171 (92.93)	13 (7.07)	0 (0.0)	355 (96.5)	13 (3.5)	1.0
c.221T>C (L74P)	170 (92.39)	13 (7.07)	1 (0.54)	353 (95.9)	15 (4.1)	0.5141
c.244G>A (E82E)	179 (97.28)	5 (2.72)	0 (0.0)	363 (98.6)	5 (1.4)	1.0
c.265A>G (T89A)	183 (99.46)	0 (0.0)	1 (0.54)	366 (99.5)	2 (0.5)	0.054
c.288A>G(P96P)	113 (61.41)	71 (38.59)	0 (0.0)	297 (80.7)	71 (19.3)	<0.001
c.294G>A (W98X)	171 (92.93)	13 (7.07)	0 (0.0)	355 (96.5)	13 (3.5)	1.0
c.296T>C (V99A)	183 (99.46)	0 (0.0)	1 (0.54)	366 (99.5)	2 (0.5)	0.0054
c.298G>A(E100K)	178 (96.74)	6 (3.26)	0 (0.0)	362 (98.4)	6 (1.6)	1.0
c.315G>A (M105I)	176 (95.65)	7 (3.8)	1 (0.54)	359 (97.6)	9 (2.4)	0.1906
c.320G>T (W107L)	156 (84.78)	26 (14.13)	2 (1.09)	338 (91.8)	30 (8.2)	0.6726
c.333C>T (L111L)	131 (71.2)	53 (28.8)	0 (0.0)	315 (85.6)	53 (14.4)	0.0249
c.336T>C (Al12A)	168 (91.3)	16 (8.7)	0 (0.0)	352 (95.7)	16 (4.3)	1.0
c.354+6C>T	137 (74.46)	46 (25.0)	1 (0.54)	320 (87.0)	48 (13.0)	0.2991
c.354+27C>T	117 (63.59)	67 (36.41)	0 (0.0)	301 (81.8)	67 (18.2)	0.0012
c.354+33G>A	35 (19.02)	138 (75.0)	11 (5.98)	208 (56.5)	160 (43.5)	<0.001
c.354+51A>G	72 (39.13)	111 (60.33)	1 (0.54)	255 (69.3)	113 (30.7)	<0.001
c.354+69C>A	116 (63.04)	51 (27.72)	17 (9.24)	283 (76.9)	85 (23.1)	0.0064
c.354+94A>G	41 (22.28)	123 (66.85)	20 (10.87)	205 (55.7)	163 (44.3)	<0.001

M/M: major homozygote; M/m: heterozygote; m/m: minor homozygote; M: major allele; m: minor allele.

**Table 2:** Linkage disequilibrium (LD) scores of the single nucleotide polymorphisms of the shadow of prion protein gene (*SPRN*) in Pekin ducks.

	1	2	3	4	5	6	7	8	9	10	11	12	13	14	15	16	17	18	19	20	21	22	23	24	25	26	27
1	-																										
2	0.021	-																									
3	0.002	0.005	-																								
4	0.17	0.003	0	-																							
5	0.003	0.06	0	0.001	-																						
6	0.002	0	0	0.05	0	-																					
7	0.014	0.017	0.017	0.004	0.003	0.003	-																				
8	0.026	0.004	0.017	0.005	0.001	0.001	0.14	-																			
9	0.234	0.061	0.009	0.04	0.013	0.001	0.006	0.111	-																		
10	0.014	0.001	0	0.002	0	0	0.002	0.008	0.009	-																	
11	0.016	0.001	0.129	0.003	0.015	0	0.007	0.001	0.023	**0.391**	-																
12	0.005	0.001	0	0.001	0	0	0.044	0.119	0.022	0.048	0.04	-															
13	0.002	0	0	0	0	0	0.002	0	0.003	0	0	0	-														
14	**0.48**	0.013	0.001	0.068	0.002	0.007	0.045	0.017	0.15	0.009	0.01	0.003	0.001	-													
15	0.014	0.001	0	0.002	0	0	0.002	0	0.008	**0.846**	**0.492**	0.048	0	0.009	-												
16	0.002	0	0	0	0	0	0.002	0	0.003	0	0	0	**1**	0.001	0	-											
17	0.006	0.001	0	0.001	0	0	0.027	0.093	0.026	0.001	0.001	0.122	0	0.004	0.001	0	-										
18	0.009	0.266	0	0.002	0	0	0.008	0.002	0.018	0.001	0.001	0	0	0.006	0.001	0	0	-									
19	0.033	0.005	0.062	0.006	0.001	0.001	0.005	0.025	0.055	0.003	0.014	0	0	0.021	0.003	0	0	0.001	-								
20	**0.423**	0.001	0.001	0.153	0.001	0.014	0.01	0.012	0.106	0.006	0.007	0.002	0.001	0.303	0.006	0.001	0.003	0.004	0.015	-							
21	0.122	0.001	0	0.003	0	0	0.014	0.003	0.029	0.002	0.002	0.001	0	0.096	0.002	0	0.001	0.001	0	0.09	-						
22	0.094	0.008	0.001	0.05	0.001	0.001	0.145	0.001	0.094	0	0.002	0.001	0.001	0.127	0	0.001	0.002	0.004	0.013	0.006	0	-					
23	**0.569**	0.012	0.001	0.153	0.002	0.008	0.006	0.016	0.113	0.008	0.009	0.003	0.001	**0.403**	0.008	0.001	0.004	0.006	0.02	0.309	0.056	0.033	-				
24	0.25	0.032	0.004	0.045	0.006	0.011	0.215	0.037	0.08	0.028	0.033	0.011	0.007	0.184	0.028	0.007	0.013	0.033	0.068	0.189	0	0.115	0.171	-			
25	**0.642**	0.024	0.002	0.143	0.004	0	0.139	0.031	0.207	0.016	0.019	0.006	0.002	**0.462**	0.016	0.002	0.007	0.011	0.022	**0.353**	0.103	0.188	**0.45**	**0.341**	-		
26	0.112	0.106	0.002	0.019	0.027	0.002	0.064	0.005	0.124	0.011	0.006	0.001	0.002	0.072	0.003	0.002	0.008	0.059	0	0.051	0.014	0.045	0.067	0.135	0.133	-	
27	0.225	0.046	0.004	0.08	0.01	0.007	0.085	0.041	0.066	0.029	0.007	0.011	0.004	0.221	0.029	0.004	0.013	0.015	0.029	0.134	0.057	0.106	0.169	**0.456**	0.242	0.125	-

1, c.9G>A; 2, c.24C>T; 3, c.39G>T; 4, c.64G>A; 5, c.78C>T; 6, c.113T>C; 7,c.164G>A; 8, c.180G>A; 9, c.183G>A; 10, c.202G>A; 11, c.221T>C; 12, c.244G>A; 13, c.265A>G; 14 c.288A>G; 15, c.294G>A; 16, c.296T>C; 17, c.298T>C; 18, c.315G>A; 19, c.320G>T; 20, c.333C>T; 21, c.336T>C; 22, c.354+6C>T; 23, c.354+27C>T; 24, c.354+33G>A; 25, c.354+51A>G; 26, c.354+69C>A; 27, c.354+94A>G. Bold font indicates strong LD (*r*^2^ > 0.333). The below diagonal indicates the *r*^2^ values.

**Table 3:** Haplotype frequencies of the 27 *SPRN* polymorphisms in Pekin ducks.

Haplotypes	Frequency, %
GCGGCTGTGGTGAAGTGGGCTCCGACA	24.3
GCGGCTGTAGTGAAGTGGGCTCCGACA	13.6
GCGGCTGTAGTGAAGTGGGCTCCAAAG	5.3
GCGGCTGTGGTGAAGTGGGCTCCGAAG	2.4
GCGGCTACAGTGAAGTGGGCTCCGACA	2.0
GTGGCTGTAGTGAAGTGAGCTCCAAAG	1.6
ACGGCTGTGGTGAGGTGGGCTCTAGCG	1.5
ACGGCTATGGTGAGGTGGGTTCTAGCG	1.3
GCGGCTGTAGTGAAGTGGTCTCCGGCA	1.3
ACGGCTGTGGTGAGGTGGGTTCTAGCG	1.2
ACGGCTATGGTGAGGTGGGCTTCAGCG	1.1
ACGACTATGGTGAGGTGGGTTTTAGCG	1.1
Others *	43.3

Others * contain rare haplotypes with <1.0%.

**Table 4:** *In silico* evaluation of the effects of non-synonymous single nucleotide polymorphisms on the Sho protein in Pekin ducks.

Amino Acid Substitutions	MutPred2	SIFT	SNPs&GO
A22T	Benign (0.045)	Deleterious (0.020)	Neutral (0.019)
M38T	Benign (0.041)	Tolerated (0.330)	Neutral (0.031)
G49D	Benign (0.043)	Deleterious (0.010)	Neutral (0.076)
A68T	Benign (0.049)	Deleterious (0.010)	Neutral (0.055)
L74P	Benign (0.112)	Tolerated (0.190)	Neutral (0.110)
E82K	Benign (0.045)	Tolerated (0.430)	Neutral (0.067)
T89A	Benign (0.029)	Tolerated (0.230)	Neutral (0.051)
W98X	Benign (0.276)	NA	NA
V99A	Benign (0.032)	Tolerated (0.510)	Neutral (0.033)
E100K	Benign (0.053)	Tolerated (0.470)	Neutral (0.059)
M105I	Benign (0.030)	Deleterious (0.030)	Neutral (0.077)
W107L	Benign (0.087)	Tolerated (0.060)	Neutral (0.126)

NA: Not applicable.

## Data Availability

All data generated or analyzed during this study are available from the corresponding author upon reasonable request.

## Supplementary Materials

The following supporting information can be downloaded at: https://www.mdpi.com/article/10.3390/ani14111588/s1. Table S1: Detailed information about the nucleotide and amino acid sequences of the shadow of prion protein gene (*SPRN*) analyzed in this study. Figure S1: Tertiary structure of the Sho protein in Pekin ducks predicted using SWISS-MODEL. (A) The tertiary structure of the duck Sho protein with the wild-type allele predicted using SWISS-MODEL. (B) The tertiary structure of the duck Sho protein with the nonsense polymorphism at codon 98 predicted using SWISS-MODEL. (C) Analysis of hydrogen bonds in the duck Sho protein with the wild-type allele predicted using SWISS-MODEL, visualized using SWISS-Pdb Viewer. Green dotted lines indicate hydrogen bonds. Helix ribbons are displayed in white and yellow. (D) Analysis of hydrogen bonds in the duck Sho protein with the nonsense polymorphism at codon 98 predicted using SWISS-MODEL, visualized using SWISS-Pdb Viewer. Green dotted lines indicate hydrogen bonds. Helix ribbons are displayed in white and yellow.
