# Supplementary material for: The First Genetic Characterization of the SPRN Gene in Pekin Ducks (Anas platyrhynchos domesticus)
Source: Animals (Basel). 2024 May 27;14(11):1588. doi: 10.3390/ani14111588 (PMC11171214; doi:10.3390/ani14111588)
Supplement: Supplementary file 1 [file animals-14-01588-s001.zip › Table S1.pdf]

Supplementary Table S1. Detailed information about the nucleotide and amino acid sequences of the *SPRN* gene analyzed in this study.

Scientific name	Common name	GenBank ID		Amino acid sequence length	Identity
		Nucleotides	Amino acids		
Homo sapiens	Human	Not applicable	NP_001012526.2	151	58/117 (49.57 %)
Bos taurus	Cattle	Not applicable	AAY83885.1	143	53/117 (45.30 %)
Capra hircus	Goat	Not applicable	AGU17009.1	146	54/117 (46.15%)
Ovis aries	Sheep	Not applicable	NP_001156033.1	145	55/117 (47.01 %)
Canis lupus familiaris	Dog	Not applicable	XP_038296952.1	147	56/117 (47. 86 %)
Equus caballus	Horse	Not applicable	XP_023492126.1	147	55/117 (47.01 %)
Gallus gallus	Chicken	BN000836.1	CAJ43796.1	117	117/117 (100 %)
Anas platyrhynchos domesticus	Duck	In this study	In this study	117	-
