# Supplementary figures and images for: The First Genetic Characterization of the SPRN Gene in Pekin Ducks (Anas platyrhynchos domesticus)
Source: Animals (Basel). 2024 May 27;14(11):1588. doi: 10.3390/ani14111588 (PMC11171214; doi:10.3390/ani14111588)

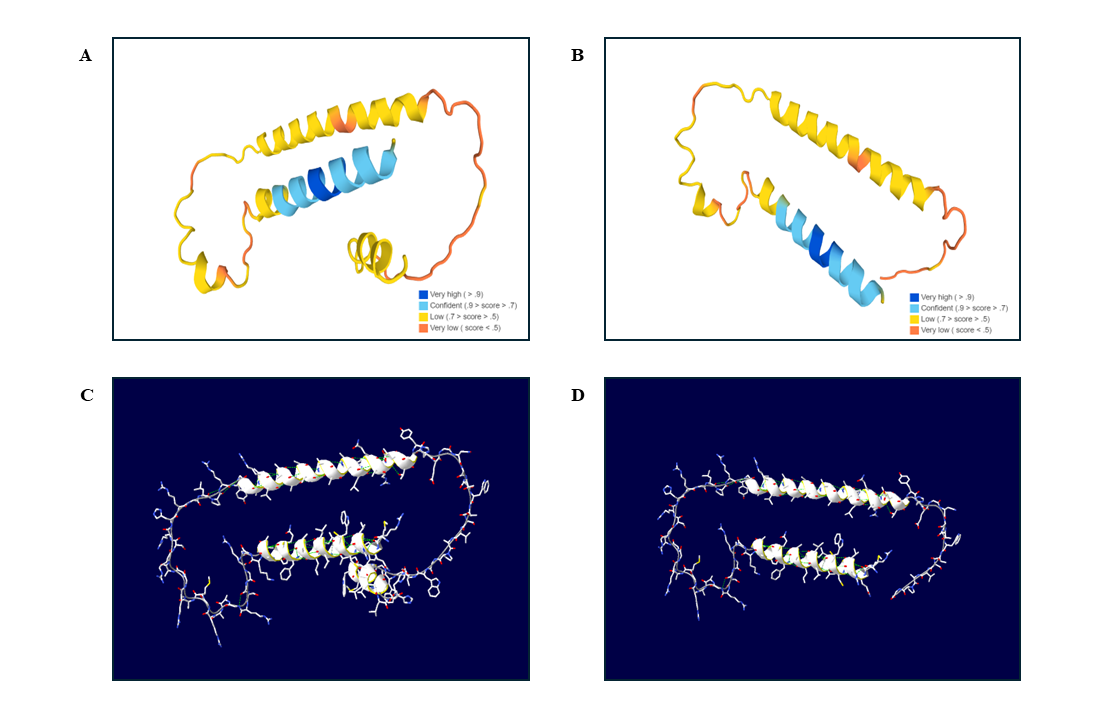

Supplement: Supplementary file 1 [file animals-14-01588-s001.zip › Figure S1.tif]
